# Screening for *Selenomonas noxia* in a Pediatric and Adolescent Patient Population Reveals Differential Oral Prevalence across Age Groups

**DOI:** 10.3390/ijerph21040391

**Published:** 2024-03-23

**Authors:** Katelyn Hendricks, Tyler Hatch, Karl Kingsley, Katherine M. Howard

**Affiliations:** 1 Department of Advanced Education in Pediatric Dentistry, School of Dental Medicine, University of Nevada-Las Vegas, 1700 West Charleston Blvd, Las Vegas, NV 89106, USA; hendrk7@unlv.nevada.edu; 2 Department of Clinical Sciences, School of Dental Medicine, University of Nevada-Las Vegas, 1700 West Charleston Blvd, Las Vegas, NV 89106, USA; hatcht3@unlv.nevada.edu; 3 Department of Biomedical Sciences, School of Dental Medicine, University of Nevada-Las Vegas, 1001 Shadow Lane, Las Vegas, NV 89106, USA; katherine.howard@unlv.edu

**Keywords:** oral screening, saliva, *Selenomonas noxia*, pediatric dentistry, adolescent oral health

## Abstract

*Selenomonas noxia*, a gram-negative anaerobe usually present in periodontitis, may be linked to overweight and obese adults. Recent advancements include a valid qPCR screening, enabling an effective prevalence study among pediatric patients aged 7 to 17 years. The aim of this study was to complete a retrospective screening of saliva samples from an existing biorepository using a validated qPCR screening protocol. The pediatric study sample (*n* = 87) comprised nearly equal numbers of males and females, mostly minority patients (67%), with an average age of 13.2 years. Screening for *Selenomonas noxia* revealed 34.4% (*n* = 30/87) positive samples, evenly distributed between males and females (*p* = 0.5478). However, an age-dependent association was observed with higher percentages of positive samples observed with higher ages (13.3% among 7 to 10 years; 34.6% among 11 to 13 years; 54.8% among 14–17 years), which was statistically significant (*p* = 0.0001). Although these findings revealed no noteworthy distinctions between males or females and minorities and non-minorities, the notable contrast between younger (7 to 10 years) and older (11 to 17 years) participants, possibly influenced by factors such as hormones and behavioral traits, will require further investigation of this patient population.

## 1. Introduction

The oral pathogenic bacteria *Selenomonas noxia* has been associated with the development and progress of periodontal disease, along with other gram-negative organisms such as *Porphyromonas gingivalis*, *Treponema denticola*, *Tannerella forsythia* and *Fusobacterium nucleatum* [1,2,3]. These bacteria are mainly associated with the development and progression of periodontitis, which has been linked with the host inflammatory response to these microbial pathogens, as well as underlying medical conditions, including diabetes [4]. The association of periodontitis among younger and pediatric patients in the United States has mainly been observed in adolescent and teenage patients with orthodontic appliances and brackets [5,6].

Members of the *Selenomonas* family are motile anaerobic bacteria typically found in the gastrointestinal tract but have also been found in periodontal pockets and other anaerobic spaces [7,8]. In particular, *S. noxia* has been found in periodontal lesions in patients with and without immunodeficiency disease, as well as those in various stages of periodontal disease development and progression—demonstrating an ability to survive within these anaerobic conditions among the many cytokines and leukotoxins secreted by other periodontal pathogens such as *Porphyromonas*, *Treponema*, *Tannerella* and *Fusobacterium* species [9,10]. Although there may be separate and more distinct functions within the gastrointestinal tract, more recent evidence now suggests that oral *Selenomonas* species may function primarily as pathobionts, existing in small numbers within healthy individuals but promoting inflammation, periodontal pocket deepening, and biofilm formation in the presence of other oral pathogens [11,12,13].

However, recent studies from this group have revealed that some pediatric patients, with and without orthodontic brackets, also harbor this organism [14,15]. This may be of particular concern as this gram-negative anaerobe typically found in periodontitis may also colonize the gastrointestinal tract and has been strongly associated with overweight and obese adults [16,17]. In fact, recent evidence has suggested that as levels of *S. noxia* increase in the oral cavity, increased levels of this organism are found in the gastrointestinal tract, allowing this microorganism to metabolize cellulose, fibers, and other “indigestible” starches and carbohydrates, thereby increasing extracted calories from dietary fiber [18,19].

Systematic reviews have shown strong evidence that gastrointestinal microbial composition may exhibit two-way relationships with overweight and obese patients, suggesting that more information regarding microbial constituents that influence caloric balance is of paramount importance [20]. Moreover, as intervention studies have demonstrated that microbial composition may be responsive to behavioral modifications such as intermittent fasting, more information will be needed regarding the species composition deemed most important to these phenomena [21]. Finally, an understanding of the additional sites that harbor this organism may provide significant insight into the relationship between oral and gastrointestinal microbial prevalence and whether other significant interventions, such as fecal transplantation, may not be feasible against *S. noxia* [22].

To aid in this quest, recent developments have led to the development of a rapid qPCR screening protocol, which has allowed for the pursuit of these initial studies involving prevalence among pediatric patients—although much remains to be discovered [23]. However, the majority of pediatric studies of these oral pathogens (including *S. noxia*) usually focus more specifically on patients with diabetes, which greatly increases the risk for the development of periodontitis [24,25]. Combined with studies of pediatric patients with orthodontic brackets, there is some information regarding the pediatric prevalence of this organism—although information regarding healthy patients without serious comorbidities or oral appliances is severely lacking [26,27,28].

Based upon the lack of evidence regarding the epidemiology of this organism, the primary goal of this project is to screen an existing saliva repository to determine the overall prevalence among the pediatric clinic population.

## 2. Materials and Methods

### 2.1. Protocol and Study Approval

This study was reviewed and approved by the University of Nevada Las Vegas (UNLV) Institutional Review Board (IRB) and the Office for the Protection of Research Subjects (OPRS) under the protocol “Retrospective analysis of microbial prevalence from DNA isolated from saliva samples originally obtained from the University of Nevada, Las Vegas (UNLV) School of Dental Medicine (SDM) pediatric and clinical population” as Research Exempt. This study involved a retrospective analysis of DNA derived from patient saliva samples from an existing biorepository and did not involve any collections or interactions with existing patients or sensitive patient-specific information under the Department of Health and Human Services (HHS) regulation 45 CFR 46 involving research exemptions.

### 2.2. Original Sample Collection

To provide full disclosure, the saliva samples stored within the biorepository were originally collected under the IRB and OPRS-approved protocol “The Prevalence of Oral Microbes in Saliva from the UNLV School of Dental Medicine Pediatric and Adult Clinical Population”. Pediatric assent from patients over the age of five years and Informed Consent from the parent or guardian was required for participation. Inclusion criteria included patients of record at UNLV-SDM, voluntary participation in the study, and Informed Consent/Pediatric Assent. Exclusion criteria included any person who was not a patient of record at UNLV-SDM, any individual who declined to participate, and any person who declined to provide Informed Consent or Pediatric Assent. Each sample was assigned a non-duplicated, randomly generated number to ensure no patient-identifying information was associated with a sample. Basic information, such as age, sex, and race or ethnicity, were the only demographic characteristics collected.

### 2.3. DNA Analysis

Saliva samples were stored in a −80 °C freezer in a biomedical laboratory. Previous research studies isolated DNA from samples using the phenol:chloroform extraction method, as previously described [15,29]. For the current study, DNA samples were identified from the biorepository and assessed using the NanoDrop 2000 spectrophotometer from Fisher Scientific (Fair Lawn, NJ, USA). In brief, absorbance values at A260 nm and A280 nm were obtained to calculate the quality and quantity of DNA within each sample. Samples from pediatric patients with greater than 10 ng and A260:A280 ratios above 1.65 were considered acceptable for inclusion in the study.

### 2.4. qPCR Screening

All samples were screened using the positive control 16S rRNA primers to ensure the presence of bacterial DNA within the sample. In addition, each sample was also screened using validated primers for *S. noxia* using the Fast SYBR green master mix from Fisher Scientific (Fair Lawn, NJ, USA). In brief, each reaction contained a 1:2 ratio of 2X Master Mix with the addition of 1.5 μL of forward and reverse primers and 2.0 μL of sample DNA. The screening was performed using the QuantStudio system from Applied Biosciences (Waltham, MA, USA) using the recommended manufacturer settings, which included 95 °C enzyme activation for 20 s, followed by 40 cycles of 95 °C for five seconds with annealing and extension at 60 °C for 30 s. Primer sequences synthesized by Eurofins Scientific (Louisville, KY, USA) included [23,30]:

Positive control 16S rRNA

16S forward primer: 5′-ACG CGT CGA CAG AGT TTG ATC CTG GCT-3′;

16S reverse primer: 5′-GGG ACT ACC AGG GTA TCT AAT-3′; Tm: 62 °C;

*Selenomonas noxia*

SN forward primer: 5′-TCT GGG CTA CAC ACGT ACT ACA ATG-3′;

SN reverse primer: 5′-GCC TGC AAT CCG AAC TGA GA-3′;

### 2.5. Statistical Analysis

Basic demographic information for each sample was compiled and presented as simple descriptive statistics. Analysis of differences between the study sample and the overall clinic population demographics was completed using Chi-Square statistics, which is appropriate for the analysis of non-parametric data [23,30]. In addition, the comparison of *Anoxia-positive* and *S. noxia*-negative samples based on demographic characteristics, such as sex and race or ethnicity, was also performed using Chi-Square analysis.

## 3. Results

A total of *n* = 87 samples were identified from the saliva biorepository for analysis in the current study (Table 1). These data indicated that nearly equal proportions of these samples were derived from males (48.3%) and females (51.7%), which was similar to the percentage of males and females in the overall pediatric clinical population, *p* = 0.8412. In addition, the proportion of samples derived from minority (66.7%) and non-minority patients was also similar to the overall pediatric clinic population, *p* = 0.0647. Finally, the average age of patients from the study sample was 13.2 years (range 7–17 years), which was significantly higher than the overall population within the pediatric clinic of 9.04 years (range 0–17 years), *p* = 0.0211.

**Figure 1:**
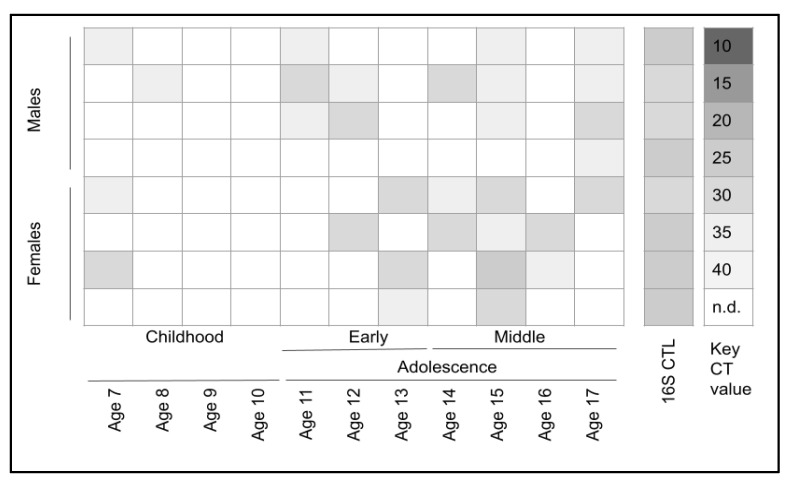
Sample screening for *S. noxia* using qPCR. A total of 34.4% (*n* = 30/87) samples harbored *S. noxia*, equally distributed among females and males (*n* = 15 each, respectively). Fewer *S. noxia*-positive samples were derived from younger children ages 7 to 10 years (13.3% or *n* = 4/30) than those from adolescents aged 11–17 years (86.7% or *n* = 26/30).

To determine if any of the selected samples from the biorepository harbored *S. noxia*, qPCR screening using validated primers was performed (Figure 1). This screening revealed that a total of 34.4% (*n* = 30/87) samples harbored *S. noxia*. Further analysis revealed that the samples testing positive for this organism were equally distributed among females and males (*n* = 15 each, respectively). However, those samples testing positive that were derived from younger children ages 7 to 10 years were significantly fewer (13.3% or *n* = 4/30) than those testing positive from adolescents aged 11–17 years (86.7% or *n* = 26/30).

To more closely evaluate these data, the demographic characteristics of the *S. noxia*-positive and negative samples were analyzed (Table 2). This analysis revealed that the percentages of males and females from the *S. noxia*-positive samples closely matched the percentages of samples testing negative, *p* = 0.5478. In addition, the percentage of samples testing positive that were derived from White/Caucasian (36.7% or *n* = 11/30) and non-White/Minority patients (63.3% or 19/30) was also similar to those testing negative, *p* = 0.2838. However, those samples derived from younger children ages 7 to 10 years were significantly lower (13.3% or *n* = 4/30) than those from adolescents aged 11–17 years (86.7% or *n* = 26/30) and were also found to be different from the samples testing negative (40.4% and 59.6%, respectively), *p* = 0.0001.

## 4. Discussion

The objective of this study was to evaluate the prevalence of *S. noxia* using an existing repository of samples derived from a pediatric clinic population. These results demonstrated that this organism is present in pediatric samples from patients as young as seven years old. This may be among the first studies to demonstrate that *S. noxia* may be present in patients this young, confirming observations from the few other studies of prevalence screenings revealing this organism among patients within similar age ranges [8,20]. In addition, this study also found a clear association with age, with younger patients exhibiting decreased prevalence and many more of the older patients harboring this organism.

In addition, this may be the first study to determine that the presence of *S. noxia* may be age-dependent within pediatric-age patient populations, with prevalence increasing significantly from the youngest age group (7 to 10 years, 13.3%) compared with early (11 to 13 years, 34.6%) and middle (14 to 17 years, 54.8%) adolescents. Although other studies have demonstrated an age-dependent association with the prevalence of this organism, these studies evaluated adults and more specifically, women during pregnancy and postpartum and elderly adults with and without periodontitis over much larger age ranges much later in life [31,32]. Based upon the lack of evidence within this very young age range, these may be among the first observations to document the age range for the acquisition of and colonization by *S. noxia* among these patients.

This may be important to consider for the oral and systemic health of children and adolescents, as many studies now confirm that the presence of this organism is associated with overweight and obesity in adults related to the metabolic capabilities of *S. noxia* to extract energy and calories from indigestible starches and carbohydrates from dietary sources [17,18,19]. Although little information is available regarding these effects in children and adolescents, a recent study revealed strong associations between increased body mass index (BMI) and other periodontal pathogens, such as *Veillonella*, *Haemophilus*, and *Prevotella*—as well as sex-specific adolescent changes in *Neisseria* and *Rothia* species [33]. Although no sex-specific associations with this organism were found in the current study, these results suggest further investigation into the association between BMI and *Selenomonas* may be warranted, given the association between this organism and obesity in adults and the associations of other oral bacteria and BMI changes in adolescents [34,35,36].

Despite the significance of these findings, this study also has some limitations that should also be considered. For example, this study utilized an existing biorepository of existing saliva samples and may, therefore, not represent an accurate representation of the most current oral health status and oral microbial composition within the pediatric patient population [14,15]. Future studies might be needed to prospectively collect samples, which may also allow for the opportunity to collect other biometric and health-related information, such as BMI and caries risk, that could help contextualize these results [37,38]. Moreover, a more detailed analysis of additional oral microbes might help with the analysis of which patients are most likely to harbor this organism and what oral health measures might be most useful to limit the distribution and spread of this organism within the oral cavity. In addition, as a retrospective study, there was no ability to review how these changes may be affected over time, such as if an early acquisition of *S. noxia* remains during adolescence or adulthood. Future studies that include a longitudinal component may help answer these questions. Furthermore, as the majority of patients within the clinic population are minority patients, other studies, including a wider variety of patients across racial and ethnic backgrounds, may provide additional information that could prove useful. Finally, future prospective studies that include the assessment of other oral health conditions, including periodontal disease or other bacterial pathogens, might provide more detailed qualitative information that could help oral health researchers determine how and why these pediatric patients may be more susceptible to acquisition and colonization by *S. noxia* as well as what the long-term effects might be [39,40,41].

## 5. Conclusions

These findings revealed no noteworthy distinctions between males or females and minorities and non-minorities within this patient population sampling. However, a notable contrast emerged between younger (7 to 10 years) and older (11 to 17 years) participants, hinting at age-related variations, possibly influenced by factors such as hormones and behavioral traits—although more exploration of these potential associations will be needed. Further investigations are required to ascertain which factors are closely linked to the presence or absence of this organism within the patient population.

## Figures and Tables

**Table 1:** Demographic analysis of study samples.

Demographic	Study Sample (*n* = 87)	Pediatric Clinic Population	Statistical Analysis
*Sex*			
Male	48.3% (*n* = 42/87)	47.2%	X^2^ = 0.040, d.f. = 1
Female	51.7% (*n* = 45/87)	52.8%	*p* = 0.8412
*Race or Ethnicity*			
White/Caucasian	33.3% (*n* = 29/87)	24.7%	X^2^ = 3.413, d.f. = 1
Non-White/Minority	66.7% (*n* = 58/87)	75.3%	*p* = 0.0647
Hispanic	31.0% (*n* = 27/87)	52.4%	
Black/African Amer.	19.5% (*n* = 17/87)	12.2%	
Asian/Pacific Island.	10.3% (*n* = 9/87)	3.8%	
*Age*			
Average	13.2 years	9.04 years	Two-tailed *t*-test *p* = 0.0211
Range	7 to 17 years	0 to 17 years	

**Table 2:** Demographic analysis of *S. noxia*-positive and negative samples.

Demographic	*S. noxia*-Positive	*S noxia*-Negative	Statistical Analysis
*Sex*			
Males	50% (*n* = 15/30)	47.4% (*n* = 27/57)	X^2^ = 0.361, d.f. = 1
Females	50% (*n* = 15/30)	52.6% (*n* = 30/57)	*p* = 0.5478
*Race or Ethnicity*			
White/Caucasian	36.7% (*n* = 11/30)	31.6% (*n* = 18/57)	X^2^ = 1148, d.f. = 1
non-White/Minority	63.3% (*n* = 19/30)	68.4% (*n* = 39/57)	*p*= 0.2838
*Age*			
Average	13.03 yrs	13.27 yrs	
Age 7 to 10	13.3% (*n* = 4/30)	86.7% (*n* = 26/30)	X^2^ = 21.275, d.f. = 2
Age 11 to 13	34.6% (*n* = 9/26)	65.4% (*n* = 17/26)	*p* = 0.0001
Age 14 to 17	54.8% (*n* = 17/31)	45.2% (*n* = 14/31)	

## Data Availability

Data from this study may be made available directly from the study author.
